# Interaction strengths in balanced carbon cycles and the absence of a relation between ecosystem complexity and stability

**DOI:** 10.1111/ele.12266

**Published:** 2014-03-17

**Authors:** Anje-Margriet Neutel, Michael AS Thorne

**Affiliations:** British Antarctic SurveyHigh Cross, Madingley Rd, Cambridge, CB3 0ET, UK

**Keywords:** Biodiversity, communities, complexity, ecological networks, ecosystems, feedbacks, food webs, interaction strength, omnivory, stability

## Abstract

The strength of interactions is crucial to the stability of ecological networks. However, the patterns of interaction strengths in mathematical models of ecosystems have not yet been based upon independent observations of balanced material fluxes. Here we analyse two Antarctic ecosystems for which the interaction strengths are obtained: (1) directly, from independently measured material fluxes, (2) for the complete ecosystem and (3) with a close match between species and ‘trophic groups’. We analyse the role of recycling, predation and competition and find that ecosystem stability can be estimated by the strengths of the shortest positive and negative predator-prey feedbacks in the network. We show the generality of our explanation with another 21 observed food webs, comparing random-type parameterisations of interaction strengths with empirical ones. Our results show how functional relationships dominate over average-network topology. They make clear that the classic complexity-instability paradox is essentially an artificial interaction-strength result.

## Introduction

**Figure 1:**
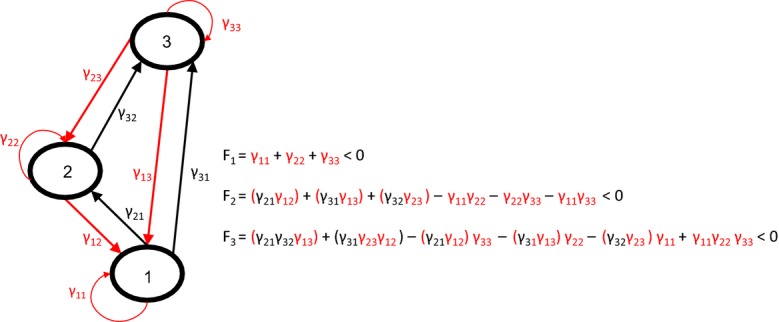
An example illustrating the relation between feedback and stability. Negative feedback must prevail, in order for a system to be stable. The diagram shows a predator-prey structure of three interacting populations. The negative effects of predators on prey (loss through predation) and the self-damping effects (loss through intragroup competition) are shown in red and the positive effects of prey on their predators (gain through feeding) are shown in black. The condition for stability that negative feedback must outweigh positive feedback at all levels is shown next to the diagram (with the brackets delineating a loop and their colour referring to the sign of the feedback loop – red for negative, black for positive). Note that in the combination a of a 2-link feedback and a self-damping effect the resulting sign is reversed (Levins 1974).

Ecosystems form intricate food webs (Pimm 1982), with cross connections between food chains, and with links from all species back to the base of the food chains, through recycling of dead organic matter (DeAngelis 1992). The immense complexity of such ecological networks contains a multitude of positive and negative feedbacks, reinforcing or dampening any disturbance (Box 1, Fig. 1). This complexity remains a fundamental challenge for ecologists wanting to analyse their stability in a mechanistic way (McCann 2000; Montoya *et al.* 2006).

**Box 1 Feedback and stability**

The term feedback refers to a basic process that is the result of mutual causal interaction: A has an effect on B, and B has an effect on A. Any change in A causes a change in B, which in turn causes a change in A, etc. If the process reinforces itself, that is, if an increase in A leads to a change in B that leads to even more of A, or a decrease in A leads to a change in B that leads to even less of A, this is called positive feedback. Positive feedbacks are forces of divergence, amplifying an initial disturbance. Negative feedback works the opposite way. It counteracts an initial disturbance. Negative feedbacks are thus forces of convergence. Chains of effects can form longer feedback loops. In a 3-link feedback loop, A affects B, B affects C and C affects A. The strength of a feedback loop is the product of the strengths of all the links in the chain. Thus, the effects are not additive, but multiplicative. One very weak link in a chain of strong links reduces the feedback considerably. If one link drops out, the whole feedback loop is broken, and the feedback is gone. A negative effect does not reduce the strength of a feedback but changes its sign.

Figure 1 describes a predator-prey structure of three interacting populations. A predator and its prey generate a negative 2-link feedback by their interaction, through the negative effect of the predator on the prey and the positive effect of that prey on its predator. The three interacting populations in this predator-prey structure generate two 3-link feedbacks, one positive (through a loop of two negative effects and one positive effect) and one negative (with two positive effects and one negative effect).

Together, the positive and negative feedback loops regulate a whole system. Whether a system is stable – whether it is able to return to its original state after a disturbance – is determined by the strength of the negative and positive feedbacks. Complex systems of *n* components comprise feedback loops of various lengths: 2-link, 3-link, 4-link, and so on, up to length *n*. The total feedback at any given level *k* is a summation of the strengths of all the feedback loops of length *k* and that of all the combinations of disjunct (non-overlapping) feedback loops of shorter length, containing *k* elements. A necessary [but not sufficient, see SI (supporting information)] condition for stability states that in a system of *n* variables the total feedback *F*_*k*_ for each level *k* in the system must be negative (Levins 1974).

For predator-prey systems, 3-link feedback loops are the shortest loops that will generate positive feedback. This positive feedback will be counteracted by negative 3-link loops but also by combinations of 2-link predator-prey feedbacks and self-damping effects, as is shown in the example of the small predator-prey system in Fig. 1.

In systems theory stability has traditionally been measured according to whether the largest real part of the eigenvalue of the matrix representing the system is negative. And within matrix theory, the two properties, stability and feedback, are combined in an elegant mathematical way, through the characteristic polynomial of the matrix. The characteristic polynomial, defined as the polynomial of which the roots are the matrix's eigenvalues, has the feature that its coefficients are summations of all the feedbacks at any given level (see Dambacher *et al.* 2003). Polynomial coefficient *a*_*k*_, in the term containing the (*n*−*k*)^*th*^ exponent, *x*^*n*−*k*^, is equivalent to *F*_*k,*_ as described above. The coefficient *a*_*k*_ is also related to the eigenvalues in being a summation of all the *k*-combinations of the different eigenvalues of the system (Brooks 2006). The signs of the coefficients determine whether the system has predominantly negative or positive feedback: if all the coefficients have the same sign, the total feedback on all levels is negative and the above condition for stability is met.

Ecosystems are traditionally modelled as Jacobian (community) matrices (May 1972; Levins 1974). These models evaluate the local stability of a system of (non-linear) differential equations, assuming a balance in the growth and loss rates of all the interacting populations. Theoretical analyses of ecosystem stability have identified relationships between the size, architecture or sign structure of ecological networks and their stability, showing a destabilising effect of species diversity or connectedness (Gardner & Asby 1970; May 1972; Levins 1968, 1974) and of omnivory (feeding on different levels in a food chain; Pimm & Lawton 1978). These topological properties have been and are still the focus of much theoretical (Chen & Cohen 2001; Dambacher *et al.* 2003; Allesina & Tang 2012) and empirical food-web research. However, the models that generate the relationships between these topological properties and system stability assume no or very little structure in the values of the elements of the community matrices, the strengths of the interactions between the populations (DeAngelis 1975).

Observational studies have shown that in natural communities most interactions are very weak (Paine 1992; Menge *et al.* 1994; Polis 1994; Wootton 1994) and the natural patterning of strong and weak interactions has been argued to enhance system stability (Yodzis 1981; de Ruiter *et al.* 1995; McCann *et al.* 1998; Emmerson & Raffaelli 2004; Rooney *et al.* 2006) and remove the negative relation between ecosystem complexity and stability (Neutel *et al.* 2002, 2007). Moreover, it has been shown that these interaction strengths generate characteristic feedback patterns (Neutel *et al.* 2002), enabling us to compare ecosystem stability in terms of their strongest 3-species positive feedback loop (Neutel *et al.* 2007).

Yet, the present empirical basis of characteristic feedback-patterns in Jacobian matrix models of complex food webs is still limited. First, the material fluxes that constitute the interaction strengths have not been observed independently but are top-down inferred from steady-state biomass observations (Lindeman 1942; Hunt *et al.* 1987; de Ruiter *et al.* 1995; Neutel *et al.* 2007). Second, the empirical evidence for these patterns comes from sub-communities and does not contain the whole ecosystem (de Ruiter *et al.* 1995; Emmerson *et al.* 2005; Neutel *et al.* 2007). Thirdly, the system components called ‘trophic groups’ usually comprise a vast richness of species, making the resolution of the food-web descriptions low (Solow & Beet 1998; Petchey & Gaston 2002).

Here we analyse two complete Antarctic terrestrial ecosystems, with a close match between species and trophic categories, in which material flows have been measured independently. We calculate the Jacobian matrices for these systems and analyse their stability mechanistically, in terms of positive and negative feedbacks (Levins 1974). With the new observations, drawing in a range of other observed food webs, we show how for complex systems the patterning of the interaction strengths dominates over general network-topological properties in controlling ecosystem stability.

## Materials and Methods

See Fig. S1 for a schematic of the analysis.

### Material-flux networks

Our study sites were two terrestrial tundra ecosystems on Signy Island, one of the South Orkney Islands in the maritime Antarctic. They were a dry moss turf community and a wet moss carpet community, here referred to as Antarctic dry and wet tundra, respectively, with relatively simple trophic structures, where the trophic groups tend towards single-species dominance (Tilbrook 1973; Davis 1981; see SI for details).

**Figure 2:**
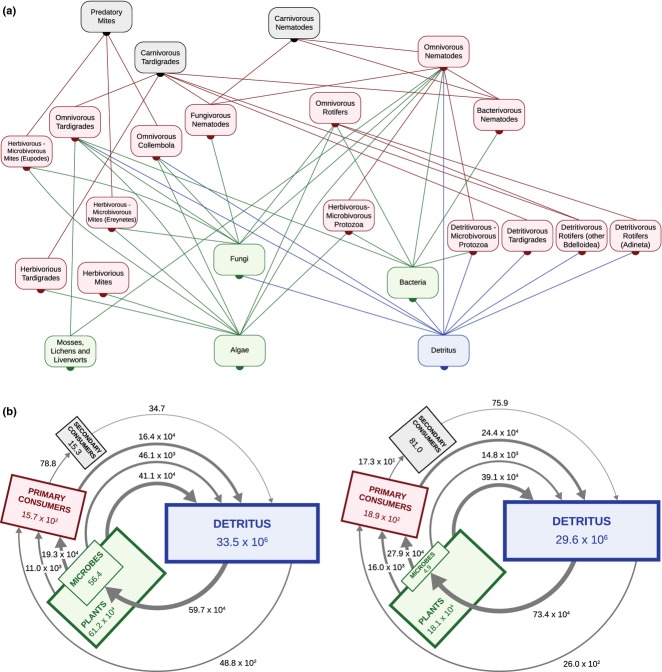
Observed material fluxes in two Antarctic ecosystems. (a) Food-web diagram of the trophic groups and feeding relations in the Antarctic dry tundra ecosystem (line colours correspond to food source). The Antarctic wet tundra has the same food web, without any mites or detritivorous-microbivorous protozoa. (b) Material cycles in the Antarctic dry (left) and wet (right) tundra approximate a balanced carbon cycle. Colours of the compartments refer to trophic levels, and correspond to those in (a). Boxes indicate total biomass (mg dry mass/m^2^), arrows the material flux (mg dry mass/m^2^y) corresponding to trophic groups in (a). Surface area of the boxes is scaled to log_10_ of the biomass.

The flow of organic matter in the systems consists of ‘upward’ transfer through feeding relations in the food chains and ‘downward’ transfer of faecal material, exuviae and carcasses from all of the organisms back to detritus (dead organic matter). We distinguished 23 trophic groups in the Antarctic dry tundra and 18 in the Antarctic wet tundra and determined the feeding relations between the trophic groups, resulting in two integrated above- and below-ground food webs (Fig. 2a).

To quantify the population sizes and fluxes in these two food webs we used independent observations of respiration rates and biomass of the species gathered over a number of years (Davis 1981). For each trophic group *j*, the annual consumption rate of each consumer was 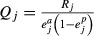, where *R*_*j*_ is the observed annual respiration rate and *e*_*j*_^*a*^ and *e*_*j*_^*p*^ are assimilation and production efficiencies respectively (Heal & MacLean 1975). Consumption rates *Q*_*ij*_ on each food source *i* were determined using observed diet specifications (Davis 1981), *Q*_*ij*_ = *f*_*ij*_*Q*_*j*_, where *f*_*ij*_ is a diet proportion (Tables S1 and S2). The other flux rates were directly related to the consumption rates, with production 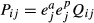, egestion 
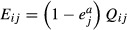
 and non-predatory mortality 
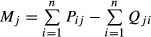
. In cases where the data were not fully specified, assumptions were made on missing values and tested for their effect on ecosystem stability (see SI for details). When we added up the non-predatory mortality rates of all the populations, the difference between this flux of dead organic matter into the detrital pool and the flux out of the detrital pool through decomposition by microbes was roughly three orders of magnitude smaller than the size of the detrital pool (Fig. 2b).

## Parameterisation of the Jacobian community matrices

Jacobian community matrices are linearisations of sets of growth equations of the populations, evaluated at equilibrium with all groups present. We followed earlier approaches and used Lotka-Volterra type growth equations (Pimm & Lawton 1978; de Ruiter *et al.* 1995; Rooney *et al.* 2006; Neutel *et al.* 2007; see SI for details). The elements of the matrices, partial derivatives of the growth equations representing the effects of the trophic groups on each other were therefore equilibrium flux rates divided by population size (with the dimension ‘per time’).

In our Jacobian community matrices **A**, effects of consumer populations *j* (predators) on their (non-detrital) food populations *i* (prey) were 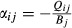, where *B*_*j*_ is the observed average biomass of *j*. Effects of prey *i* on their predators *j* were 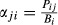. This meant that there was a direct relation between effects of predators on prey and vice versa (Pimm & Lawton 1978): 
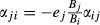
, with *j* the predator, *i* the prey and 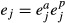. Intraspecific (competition) effects of the populations are non-predatory loss rates that are density dependent, relative to population size. The observations provided the total non-predatory mortality rates *M*_*j*_. Thus, they determined an upper bound (in absolute value) for these elements: 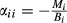. Effects on detritus were calculated from the non-predatory mortality and egestion rates obtained from observation, using a modified Lotka-Volterra equation for detritus (DeAngelis *et al.* 1989; de Ruiter *et al.* 1995). Effects of trophic groups *j* on detritus *D* were 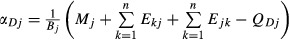. Detrital diagonal elements were fixed by the flows: 
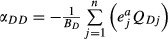
.

## Normalisation of the Jacobian community matrices

The standard method to evaluate system stability is to determine the eigenvalues of the Jacobian matrix. If all the eigenvalues have negative real parts, the system is stable, that is, it will return to the equilibrium after a perturbation of any of the state variables. Traditionally, the speed of return to its original state, measured by the size of the largest real part, is used to quantify the relative stability of systems. But this metric is dependent on the time scales of the individual populations. To avoid this time-dependence, to compare different ecosystems, the diagonal of the matrix has been proposed as a control parameter, using the relative self-damping of the populations (*s*) needed for stability (that is, for all eigenvalues to have negative real parts) as a metric to evaluate stability (Neutel *et al.* 2002). We obtained the same effect by normalising **A** and used the maximum real part of the eigenvalues (*λ*_*d*_) directly as a stability metric. Each matrix **A** was normalised by dividing each row by the absolute value of its respective diagonal element, 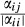, which resulted in time-independent and dimensionless matrices **Γ**.

As a result of this normalisation, effects of predators *j* on their (non-detrital) prey populations *i* became 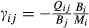, effects of prey *i* on their predators *j* became 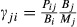, and effects of populations *j* on detritus *D* became 
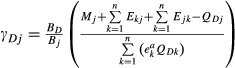
. Normalisation preserved the essential stability properties of the matrices, in the sense that the minimum level of competition needed for stability (*s*) remained on the whole unchanged (Table S3). It must be noted that our results did not depend on the normalisation and we could have used the earlier proposed metric *s*. The advantage of the normalisation step was that the mathematical analysis of the matrices became more transparent, allowing us to use the coefficients of the characteristic polynomial of the matrices to analyse stability in terms of the feedbacks in the systems. The preservation of *s* after this normalisation procedure was an empirical result and is not a general property of matrices. The success of the normalisation for the 43 systems that we studied opens an interesting research question as to how we can analytically understand this.

## Independently measured fluxes vs. inferred fluxes

### Inferred material fluxes

In order to assess the effect of using independent measurements of material fluxes on the stability of the Antarctic dry and wet tundra, we compared it with the previously used method of calculating fluxes from the steady-state assumption (Lindeman 1942; Hunt *et al.* 1987). For this, we used only the observed biomass densities, diet information and efficiencies (see SI for details). The rationale of this indirect method is that since growth equals loss at steady state, one can calculate the feeding rate of a population if its loss rate and the efficiency with which it converts its food to growth (biomass production) are known. The loss rates of a population depend on the predation rates of the populations feeding on it. Only the top predators do not experience predation. If one knows the natural (non-predatory) loss rate of a top-predator population, assumed to be roughly determined by the natural life span of the organism and the biomass of the population, one knows its growth rate and, with a given conversion efficiency, its feeding rate on the populations below. Information on the relative feeding on various prey types then allows for a top-down calculation of all the feeding rates. With the inferred material flux networks we calculated Jacobian community matrices and normalised them, following exactly the same procedures as described above (Fig. S2).

### Calculation of stability

We calculated stability of the normalised matrices **Γ** by setting all the diagonal elements to zero apart from the detrital diagonal element, which meant that the only self-damping in these matrices was provided by detrital flows with no self-damping provided by the populations (**Γ**_**0**_^**D**^). We then determined the maximum real part of the eigenvalues (*λ*_*d*_) of **Γ**_**0**_^**D**^. By determining *λ*_*d*_ we measure the vulnerability of the ecological network in the absence of self-damping competition.

In the Antarctic tundra ecosystems and their inferred-flux counterparts, with the one non-zero diagonal element resulting from detritus interactions, *λ*_*d*_ closely matched the relative self-damping of the populations (*s*) needed for stability (see SI Methods; Fig. S3) and can therefore be interpreted as the competitive ability of the populations needed for system stability. Thus, *λ*_*d*_ has a biological meaning. It shows the biological tipping point: if the intraspecific losses of the populations were to drop below a level indicated by *λ*_*d*_, the populations would not generate enough self-damping feedback and the ecosystem would collapse. Vulnerability *λ*_*d*_ = 1 refers to the maximum level of self-damping feedback, given the energetic constraints on the system. If *λ*_*d*_ > 1, more than the total non-predatory losses would come from intraspecific competition to provide stability, which means that the observed balance in material flow would not be sustainable; it would require external inputs to compensate for these losses.

## Effect of recycling on ecosystem stability

We tested the effect of recycling interactions on the vulnerability of the Antarctic tundra webs by comparing *λ*_*d*_ of **Γ**_**0**_^**D**^ with *λ*_*d*_ of the same matrices without detritus interactions (**Γ**_**0**_), the latter obtained by removing the detrital row and column from the matrices. An increase in *λ*_*d*_ after removal of detrital interactions meant that the feedbacks resulting from recycling of organic matter (detritus feedbacks) had a stabilising effect on the ecosystem, a decrease meant that they were destabilising and if *λ*_*d*_ did not change, this meant that they had no effect on system stability.

We then drew in a set of 39 soil food webs and calculated the effect of detritus in these webs. The Jacobian community matrices of these soil food webs were parameterised using inferred fluxes and then normalised as described above (see SI for details).

## 3-link positive and 2-link negative feedback predict stability

We continued our analysis with the Antarctic tundra ecosystems and 21 soil food webs that shared their property that stability was not affected by detritus, only by predator-prey interactions. To study how predator-prey feedbacks determined system stability, we removed detrital interactions and set all the diagonal elements at zero (**Γ**_**0**_). We then examined the feedbacks in the systems through the coefficients of the characteristic polynomial (Box 1). In the matrices **Γ**_**0**_, without any self-damping feedback, the second coefficient, *a*_*2*_, represented the sum of all the 2-link feedbacks, resulting from the pairs of predator-prey interactions, which are by definition negative: 
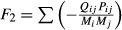
, where *i* is the prey and *j* is the predator and all parameters are defined positive. The third coefficient, *a*_*3*,_ represented the sum of all the 3-link loops, coming from the smallest omnivorous interactions, each generating a positive and a counteracting negative effect. We found that the sum of all 3-link loops was by definition positive, because of the net positive 3-link feedback from each omnivorous interaction: 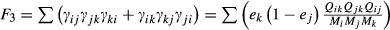, where *i* is the bottom prey, *j* is the intermediate predator, *k* is the omnivore, and *e*_*j*_ is the biomass conversion efficiency, by definition between 0 and 1. The expression 
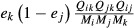
, by definition positive, results from the direct relation between the effect of a prey (*i*) on its predator (*j*) and that of the predator on its prey: 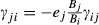.

These 2-link and 3-link loops are crucial because they play a role not only in determining *F*_*2*_ and *F*_*3*_, but also in the higher-level feedback, through combinations of feedback loops. The cube root in the relation between *λ*_*d*_ and 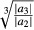 originates from the direct relation between the product of three eigenvalues and *a*_*3*_ (Brooks 2006).

### Symmetry, asymmetry, complexity: Empirical vs. synthetic parameterisation

We compared the empirically based parameterisation with standard theoretical parameterisations by constructing two sets of community matrices for each of the Antarctic systems and the 21 soil food webs, using **Γ**_**0**_**.** In the first, the non-zero values in the observed matrix were replaced by values randomly drawn from uniform distributions [−1,0) for effects of predators on prey and (0,1] for effects of prey on predators, following May (1972) but preserving the sign structure of the observed systems. In the second, the elements were chosen in an identical way, but with an asymmetry, [−10,0) and (0,0.1), following Pimm & Lawton (1978). We called the first random-type parameterisations ‘symmetric’ and the second ‘asymmetric’. The resulting vulnerability *λ*_*d*_ was the mean of the maximum real parts of the eigenvalues from 100 sampled matrices.

## Results

### Independently measured fluxes vs. inferred fluxes

We quantified the two entire above-and below-ground food webs of the Antarctic dry and wet tundra ecosystems (Fig. 2a) including recycling through dead organic matter and found that the overall material flow in both systems approximated a balanced carbon cycle (Fig. 2b).

The material fluxes together with the observed biomass densities of the populations provided us directly with the element values of the Jacobian community matrices, including the structure of the diagonals. We translated the observed structure of the diagonals into the off-diagonal elements and evaluated the stability properties of the ecosystems without assuming any self-damping through intraspecific losses (competition) by the populations. The food web of the Antarctic wet tundra was substantially more vulnerable than that of the Antarctic dry tundra, *λ*_*d*_ = 0.26 and *λ*_*d*_ = 0.04 respectively. We compared the community matrices based on the independent material-flow observations with matrices of the Antarctic systems where interaction strengths were calculated from inferred material fluxes deriving them from the steady-state assumption (Fig. S2). System stability of the Antarctic webs was remarkably similar to that of their inferred-flux counterparts (*λ*_*d*_ = 0.24 for the inferred wet and *λ*_*d*_ = 0.08 for the inferred dry tundra). This indicated that the indirect method of inferring fluxes from steady-state biomass observations reflected the direct observations sufficiently to capture the essential stability properties. We tested the robustness of the results by sampling feeding rates and biomass from the observed variability in these values (Fig. S4).

### Effect of recycling on ecosystem stability

**Figure 3:**
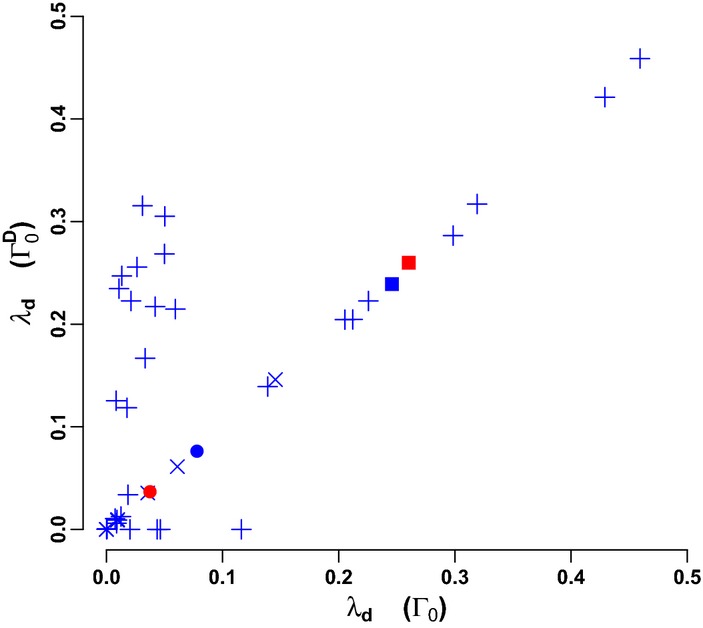
Effect of recycling on ecosystem stability. Ecosystem vulnerability *λ*_*d*_ with detritus interactions present (Γ_0_^D^) compared with system vulnerability after detritus interactions were removed (Γ_0_). The Antarctic dry tundra (closed circle) and wet tundra (closed square) are shown in red. Their inferred-flux counterparts are shown in blue, as well as the 39 soil food webs which are all based on inferred fluxes. The soil food webs are from agricultural and native soils (cross signs) (Hendrix *et al.* 1987; Hunt *et al.* 1987; Andrén *et al.* 1990; de Ruiter *et al.* 1993) and from soils in two vegetation successions (plus signs) (Neutel *et al.* 2007). For details see Table S3. Regression analysis: *N* = 43, *R*^2^ = 0.47, *P *< 10^−6^; regression analysis with only the systems where stability was not affected by detritus interactions: *N* = 25, *R*^2^ = 0.99, *P *< 10^−15^.

To investigate what determined the vulnerability of the Antarctic ecosystems, we first tested the effect of recycling of organic matter on the stability of the systems. We found that removing the recycling effects (interactions between populations and detritus) neither increased nor decreased the stability of the systems: they had no effect on system stability. We explored the generality of this result by drawing in 39 soil food webs with parameter values derived from steady-state biomass observations. Two categories of systems emerged: for 21 food webs recycling had no or a negligible effect on system stability, for 18 food webs recycling had a clear effect, either stabilising or destabilising the system (Fig. 3). It is worth noting that the latter were all communities from relatively young soils, low in organic matter, or with relatively simple network structure (Table S3). We then continued our analysis with the Antarctic matrices, their inferred-flux counterparts and the 21 soil food-web matrices that shared the property that only predator-prey feedbacks (feedbacks without interactions with detritus) affected system vulnerability.

### 3-link positive and 2-link negative feedback predict stability

Examination of all the feedback loops in the systems confirmed earlier findings that longer loops tended to be relatively weak (Neutel *et al.* 2002, 2007; Emmerson *et al.* 2005). The coefficients in the characteristic polynomials of the matrices also tended to be relatively small (in absolute value) on higher levels. The signs of the coefficients showed that our systems, with no self-damping by the populations, were systems of predominantly positive feedback. If we increased the level of self-damping up to the tipping point at which there was just enough self-damping for stability, this was also the point where positive feedback ceased to be predominant and negative feedback prevailed. Thus, we concluded that the stability of our systems depended on the balance of positive and negative feedbacks, not on the absence of time lags caused by excessive negative feedback (see SI for details). We found that the vulnerability of the systems could roughly be predicted by the net positive feedback resulting from the 3-link omnivorous interactions and the negative feedback from the 2-link predator-prey interactions according to 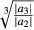, where *a*_2_ and *a*_3_ are coefficients of the characteristic polynomial of the community matrix (Box 1; Fig. S3).

This can be understood as follows. For a system to be stable, the sum total of feedback of every length has to be negative. The net positive feedback of the 3-link loops can only be compensated for by the negative feedback resulting from combinations of pair-wise predator-prey feedback and self damping effects. The stronger the 2-link negative feedback, the less self-damping needed. One could think of the positive 3-link feedback as the elementary destabilising force in trophic networks, balanced by the stabilising feedback from 2-link loops and self-damping.

### Symmetry, asymmetry, complexity: Empirical vs. synthetic parameterisation

**Figure 4:**
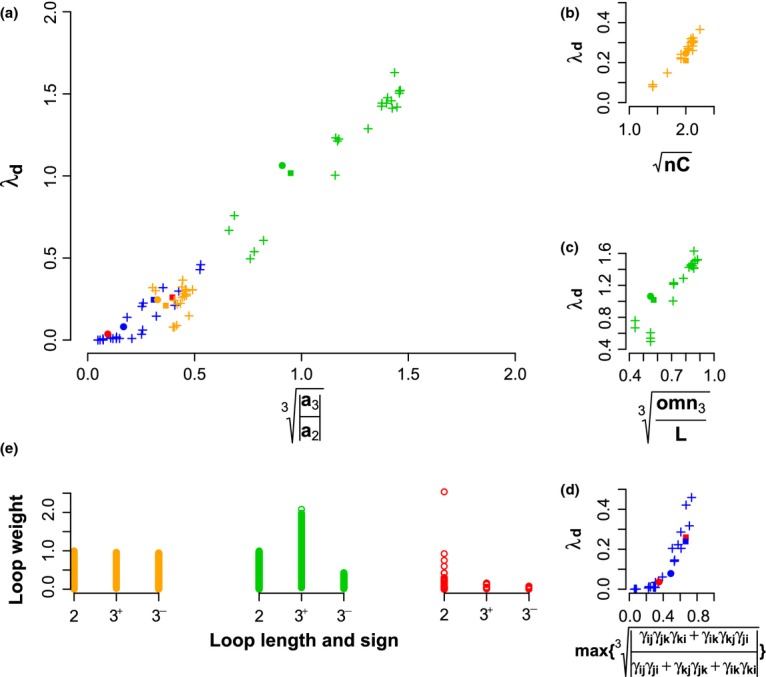
Feedbacks and stability. Empirical parameterisations of the interaction strengths of the Antarctic dry and wet tundra systems and 21 soil food webs (for legend see Fig. 3, with plus signs for the 21 soil webs) are compared with symmetric (yellow symbols) and asymmetric (green symbols) parameterisations. (a) 3-Link and 2-link predator-prey feedbacks 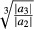 and system vulnerability *λ*_*d*_ (*N* = 71, *R*^2^ = 0.97, *P *< 10^−15^). λ_*d*_ is correlated with (b) complexity 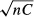 in the symmetric webs (*N* = 23, *R*^2^ = 0.94, *P *< 10^−13^), (c) the level of omnivory 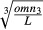 (*N* = 23, *R*^2^ = 0.84, *P *< 10^−9^) in the asymmetric webs, and (d) maximum ratio of 3-link and 2-link feedback in omnivorous structures (*N* = 25, *R*^2^ = 0.79, *P* < 10^−08^) in the empirical webs. (e) Weights (Neutel *et al.* 2002) of 2- and 3-link feedback loops in the Antarctic dry tundra for the different parameterisations. The spectra are representative of the other food webs and the symmetric and asymmetric spectra contain the 2- and 3-link feedbacks of 100 samplings of those parameterisations.

The absence of an effect of detritus feedbacks on system stability allowed us to make a direct comparison between the empirically-based parameterisations and synthetic (symmetric and asymmetric) parameterisations underlying the canonical ideas on the destabilising effects of diversity or link density (Gardner & Asby 1970; May 1972) and omnivory (Pimm & Lawton 1978). The ratio 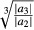 roughly predicted system stability irrespective of the type of parameterisation (Fig. 4a).

However, 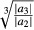 did not capture the difference between the symmetric webs. The feedback spectrum of the symmetric webs explained why this was the case (Fig. 4e). In the symmetric webs, the lack of structure in the interaction strengths created a certain homogeneity at system level, a symmetry in feedback strength between positive and negative feedback loops of the same length. This balance between positive and negative 3-link loops meant that it was just the 2-link loops that governed stability. But instead of contributing to stability, as in the asymmetric and empirical parameterisations, the 2-link predator-prey loops showed a negative relationship with system stability. The number of 2-link loops is equal to the number of interactions in the system and we found indeed that the number of links per species (degree of the network), represented by the classic complexity metric 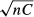 (May 1972), where *n* is the number of species and *C* is the connectance, was strongly correlated with the vulnerability of the symmetric webs (Fig. 4b).

In the asymmetric webs, the negative effects of predators on prey were much stronger than the positive effects of prey on predators. Thus, the strength of a feedback loop depended on the proportion of negative and positive effects in that loop, so that, on average, positive 3-link loops (containing two negative effects and one positive effect) were much stronger than negative 3-link loops (Fig. 4e). This made the asymmetric webs very vulnerable. In these webs, 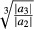 was effectively determined by the number of 3-link loops relative to 2-link loops, since values for positive and negative interactions were all randomly sampled from the same two intervals. The degree of omnivory (Pimm & Lawton 1978), here calculated as 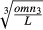, where *omn*_*3*_ is the number of 3-link omnivorous structures and *L* is the total number of interactions, was strongly correlated with the vulnerability of the asymmetric webs (Fig. 4c).

The empirically-based webs also showed an asymmetry between predator-prey and prey-predator effects (Fig. S2), but the patterning in these effects resulted in a totally different feedback spectrum (Fig. 4e). We found that the maximum of 3-link vs. 2-link feedback in omnivorous units of three interactions was strongly correlated with system vulnerability (Fig. 4d). In terms of material-flow parameters these feedback units could be expressed as:
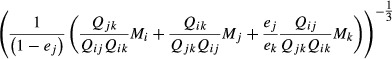
(1)where *i* is the bottom prey, *j* is the intermediate predator, and *k* is the omnivore. *Q*_*ij*_ and *M*_*j*_ are rates of consumption and non-predatory mortality respectively, and *e*_*j*_ is a biomass-conversion efficiency. All parameters are defined positive. The functional relationships expressed in (1) imply that the stronger the predation pressure (mortality due to predation relative to non-predatory mortality) in ecosystems, the stronger the critical feedback is, and hence the more competition is needed to preserve stability (see SI for details). In the Antarctic wet tundra, with a more rapid turnover of organic matter, predation pressure was much stronger than in the Antarctic dry tundra, also reflected by the larger biomass on the higher trophic levels (Fig. 2b). This explains why the Antarctic wet tundra was more vulnerable than the Antarctic dry tundra. Expression (1) confirms earlier work on the destabilising effect of strong predation (Neutel *et al.* 2007; Rip & McCann 2011), the stabilising role of both asymmetric predation rates (Neutel *et al.* 2002) and lower efficiencies (Holt & Huxel 2007) of omnivores.

## Discussion

Our study gives new direct evidence for the key role of interaction strength in the stability of ecosystems. It presents an analysis of the mathematical stability properties of two Antarctic ecosystems where almost all the fluxes in the entire above- and below-ground food web are known. The extension of the analysis to a larger set of observed ecosystems provides several insights into the effects of detrital vs. predator-prey interactions on stability and of pair-wise predator-prey feedback vs. longer feedback loops. The results shed a light on the crucial role of interaction strength in the biodiversity-stability debate.

### Independently measured fluxes vs. inferred fluxes

The detailed observations of the two Antarctic ecosystems add a level of realism when compared with previous quantifications of trophic networks, in which the method generated a material-flux balance by calculating consumption to match loss rates, with all fluxes ultimately depending on assumed turnover rates of the top predators (Lindeman 1942; Hunt *et al.* 1987; de Ruiter *et al.* 1995; Neutel *et al.* 2007). It must be emphasised that quantifying the energy flow in observed food webs has been done in many ways, for example through stable isotopes (Post 2002) or in the application of other balancing approaches, mainly used in marine ecosystems (Pauly *et al.* 2000). The difference between our study and these approaches is that we observed a whole balanced ecosystem (we did not have to perform any parameter tuning) as well as the detail and rigour with which we could test our assumptions with respect to any missing information or uncertainty of the observations.

Our results make very clear that the stabilising patterning of interaction strengths identified by earlier studies (de Ruiter *et al.* 1995; Neutel *et al.* 2007) does not depend on the method used by these studies to infer fluxes. Moreover, our stability results lend independent support for this indirect method of deriving interaction strengths from steady-state observations, by showing how the independently measured and inferred fluxes lead to almost the same stability for the Antarctic ecosystems.

### Effect of recycling on ecosystem stability

Recycling of organic matter, so important in the growth and sustainability of ecosystems (DeAngelis *et al.* 1986; Lenton & Watson 2011), generated many strong feedbacks in the Antarctic wet and dry tundra systems, but they turned out to have no impact on the ability of the systems to return to their original states after a disturbance. The observed neutrality of detritus feedbacks to stability in these systems and the large set of soil food webs raises the question of whether this is a general feature of ecosystems, and how this could be explained. A stabilising impact of detritus self-damping has been shown for simple food chains (Moore *et al.* 1993, 2004; Neutel *et al.* 1994). As for its role in the dynamics of larger networks, it is known that detritus has a slowing-down effect on the resilience of a system, through the slowing down of processes (DeAngelis 1992). We suggest that if the buffering capacity of detritus, particularly strong in older, organic-rich soils, gets threatened, not only may this speed up processes, but also the neutrality of detritus feedbacks may be lost, causing a regime shift where detritus then will affect the ability of the whole system to recover from perturbations (Fig. 3).

### 3-link positive and 2-link negative feedbacks predict stability

Our findings explain why 3-link feedback loops play a key role in ecosystem stability (Neutel *et al.* 2007), but they also show why we should take into account the combination of positive 3-link feedback and negative 2-link feedback to estimate system stability. The proposed relation between 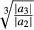 and the level of self-damping necessary for stability offers a way to test this metric empirically, by measuring the competitive ability of species. Competition within species or populations is a property central to biology, although measuring this property in food webs is difficult (but see Schmitz 1997).

The principle underlying our metric, that the relative strength of the shortest positive feedback loops and counteracting negative feedback loops governs stability, will also have implications for other types of interaction. For example, in networks with mutualism (+,+) or interference competition (−,−) the shortest positive feedback loops are 2-link loops, in which case *a*_*2*_ instead of *a*_*3*_ may be expected to be the key destabilising force.

### Symmetry, asymmetry, complexity: empirical vs. synthetic parameterisations

**Figure 5:**
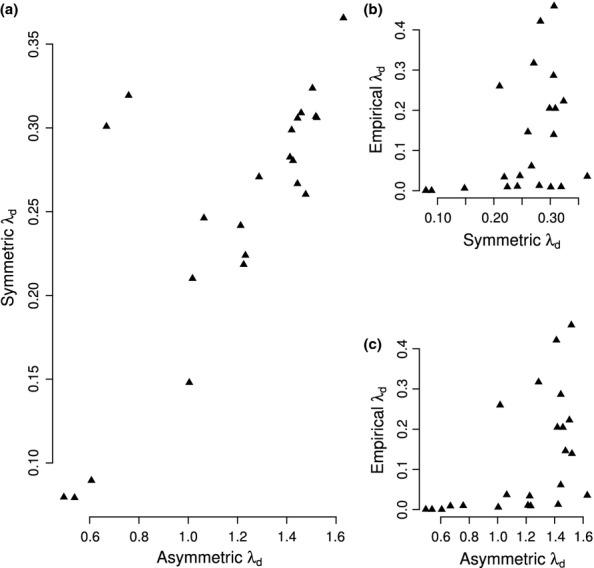
Absence of a relation between ecosystem complexity and stability. Correlation of system vulnerability *λ*_*d*_ between webs with symmetric and asymmetric interaction-strength paramerisations (a) and the absence of a correlation with either of the two, when interaction strengths are empirically based (b and c), for the two Antarctic ecosystems and the 21 soil food webs. (a) While symmetric parameterisation leads to a relation between complexity (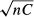) and vulnerability (Fig. 4b), asymmetric parameterisation leads to a relation between omnivory and vulnerability (Fig. 4c). Usually, more complex webs tend to be more omnivorous, hence the correlation, but there are exceptions (*N* = 23, *R*^2^ = 0.54, *P *< 10^−04^). When interaction strengths are empirically based, provided there is some basic level of complexity, the density of interactions no longer controls system stability. This explains the absence of a correlation between emprically and symmetrically (b) or asymmetrically (c) parameterised webs (*N* = 23, *R*^2^ = 0.13, *P* = 0.048 and *N* = 23, *R*^2^ = 0.24, *P *=* *0.01, respectively).

The complexity-instability paradigm in ecology suggests that ecosystem stability can be understood in terms of the density of pair-wise interactions (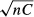) in an ecological network (Gardner & Asby 1970; May 1972). Food-web theory has long focussed on network architecture and topology to reconcile the problematic relationship between this complexity and stability. However, our results show that even in models with sign structure and topology based on observation, we do not have to go beyond the pair-wise predator-prey interactions to capture the stability of a whole network, if interaction strengths are parameterised randomly from the same (symmetric) intervals. Introducing asymmetry between top-down and bottom-up effects adds crucial realistic structure to the interaction strengths. It means we have to go one step further, to 3-link feedbacks, in order to understand whole-network stability. But a simple asymmetry overestimates the importance of the amount of omnivorous interactions in system vulnerability (Gellner & McCann 2012). As a consequence, even asymmetric parameterisations do not fundamentally challenge the view that complexity begets instability, since more omnivorous systems often tend to be more highly connected. Indeed we find a strong correlation of the stability between the symmetric and asymmetric webs (Fig. 5a). In contrast, the webs with the empirical parameterisations of interaction strengths show no correlation with either the symmetric or asymmetric webs (Fig. 5b and c). This is a result of the patterning of the interaction strengths, not just the average strength or the frequency distribution of these strengths. Observation-based interaction strengths completely remove the relation between the density of pair-wise interactions and system stability.

It has been suggested (Dambacher *et al.* 2003) that approaches that include interaction strength are complementary to approaches focusing on network architecture and sign structure. Our results show that they are not. Ecological stability analyses with synthetic parameterisation of interactions lead to specific patterns in feedback strength resulting in artificial structure-stability relationships. This shows that we have to be careful basing structural comparisons on simple synthetic parameterisations (DeAngelis 1975).

The importance of the complexity of communities for the functioning of ecosystems is well known (Hooper *et al.* 2005). Our results show mechanistically how it is only through its effect on the functional relationships, shaping the flow of matter and nutrients, that complexity may affect ecosystem stability, not through the overall topology of the network. Understanding this functional complexity should be a priority in the study of our increasingly threatened ecological systems.
