# Supplementary material for: Interaction strengths in balanced carbon cycles and the absence of a relation between ecosystem complexity and stability
Source: Ecol Lett. 2014 Mar 17;17(6):651–61. doi: 10.1111/ele.12266 (PMC4285907; doi:10.1111/ele.12266)
Supplement: Supplementary file 2 — Supplementary [file ele0017-0651-SD2.pdf]

Supporting Information

Interaction strengths in balanced carbon cycles and the absence of a relation between ecosystem complexity and stability

Anje-Margriet Neutel & Michael A.S. Thorne

This PDF file includes:

- Methods
- Tables S1 to S3
- Figures S1 to S5
- References

Methods

Study sites

The two Antarctic ecosystems consist of a relatively dry moss turf community (dry Antarctic tundra) dominated by *Polytrichum alpestre* and *Chorisodontium aciphyllum*, and a wet moss carpet community (wet Antarctic tundra) dominated by *Calliergon sarmentosum* and *Cephaloziella varians* with patches of *Drepanocladus uncinatus* and *Calliergidium austro-stramineum* (Tilbrook 1973). The trophic structure of these Antarctic tundra communities is relatively simple: there are no vascular plants, annelids, molluscs, winged insects, or terrestrial vertebrates, and the trophic groups tend towards single-species dominance (Table S1). The terrestrial vertebrates that occasionally visit the sites (seabirds, penguins and seals) all depend on the sea for their food. Even though they may have an effect on the communities in adding nutrients, they themselves are not affected by the communities. Therefore, there is no feedback and they can be considered to be not part of the system, an environmental factor.

Material-flux rates

Respiration rates were measured independently for each trophic group with various methods, from field data from the two sites. Care was taken to translate actual rates to annual rates using site specific information on temperature and active periods. Measurements of the respiration rates of the nematodes (Maslen 1981), mites (Goddard 1977a,1977b,1977c), collembola (Tillbrook 1977), tardigrades (Jennings 1976), rotifers (Jennings 1975,1976), protozoa (Smith 1973a,1973b) and micro-flora (Wyn-Williams, 1979), spanning roughly a decade of disparate studies on the ecology of the Antarctic dry and wet sites, were assembled and in some of the cases refined by Davis (1981). See Davis (1981) for more detail.

In the calculation of feeding rates (Table S1) from the respiration rates, generalised values for assimilation efficiencies, e^a_j, and production efficiencies, e^p_j, were used (Heal & MacLean 1975, Hunt *et al.* 1987). The values of the efficiencies are $e^a_j = 0.8$ and $e^p_j = 0.3$ for the three carnivorous

top-predators (predatory mites, carnivorous mites and carnivorous tardigrades), $e_j^a = 1$ and $e_j^p = 0.4$ for the detritivorous-microbivorous protozoa, bacteria and fungi, and $e_j^a = 0.3$ and $e_j^p = 0.4$ for the other consumers.

The feeding rate of each consumer which fed on several prey groups was then partitioned to obtain the feeding rates on each food source of that consumer (Table S2). The proportion of each food source in a consumer′s diet was derived from gut-content and fecal-pellet analysis in the collembola, from examination of the feeding apparatus in the nematodes and tardigrades and from the literature in the rotifers and protozoa (see Davis 1981). Specific assumptions were made and tested for their effect on system stability in cases where the proportions could not be determined from the data provided by Davis (see below).

Specific assumptions on material-flux rates

A total feeding rate of the three rotifer populations was obtained from observation (Davis 1981). We partitioned this rate between the rotifer groups according to their proportion in total rotifer biomass. Randomising this partitioning did affect feasibility (that is, it could lead to population growth rates not compensating for loss rates), but when rates were feasible, ecosystem stability was not affected.

Total biomass and total feeding rate of the three primary consumer tardigrade populations were obtained from observation (Davis 1981). We partitioned biomass and feeding rate equally between the tardigrade groups. Biomass assumptions had no effect on either feasibility or stability (Fig. S3). Randomising the partitioning of feeding rates did affect feasibility, but if feasible, stability was not affected.

Total microbial biomass and feeding rate based on aerobic activity were provided by Davis (1981). The ratio of fungal to bacterial biomass (1/10) was obtained from Bokhorst (2007). We partitioned the aerobic feeding accordingly in proportion to the biomass. Since the Antarctic wet tundra was extensively anaerobic, we added a value to the bacterial feeding rate in this ecosystem, to make bacterial feeding rates feasible. Variation of the fungal biomass (with the fungal/bacterial ratio lying between 1/50-1/5) and its corresponding feeding rate did not affect feasibility or stability.

The basal trophic groups do not feed on other trophic groups. Therefore no feeding rates are given for the primary producers in Table S1. However, the production rates of these groups are relevant for calculating the community matrices since they determine the natural mortality (see Methods in main text). On the Antarctic dry and wet tundra, the production of mosses, lichens and liverworts was 409 (321-497) g DM m^{-2} y^{-1} and 392 (226-548) g DM m^{-2} y^{-1} respectively. Algal production was not based on directly measured rates (Davis 1981). Davis provides values of 13.4 g DM m^{-2} y^{-1} for the dry tundra and 16.8 g DM m^{-2} y^{-1} for the wet tundra. For the Antarctic wet tundra we assumed a value for algal production to compensate for loss through herbivory since the value assumed by Davis resulted in unfeasiblity of algal production. Varying the values for algal production in either of the Antarctic tundra systems, provided the rates resulted in feasible systems, did not affect stability.

In the case of omnivorous nematodes, diet proportions were obtained from feeding preferences of that group observed by Hunt *et al.* (1987). In the other cases where proportions were not provided by the data, feeding was assumed to be proportional to the biomass of the food source, that is, it was assumed to be determined by prey availability (Hunt *et al.* 1987). We tested the sensitivity of our assumptions on diet preferences not provided by Davis (1981) for their effect on ecosystem stability. None of these assumptions affected stability (provided the resulting rates were feasible) apart from the assumptions on the proportions of different animal prey in the diets of carnivorous nematodes and omnivorous nematodes. In both cases, randomising prey preferences increased stability, in the same way for the Antarctic dry and wet tundra. The relative difference between the dry and wet tundra did not depend on the assumptions on diet specifications in both systems.

Determination of material-flux rates for the inferred-flux community matrices

To assess the effect of our direct method of obtaining interaction strengths with the previously used indirect method, we inferred material fluxes from the steady-state assumption, using the observed biomass values, diet specifations and the generalised values for assimilation and production efficiencies from Davis (1981) and natural (non-predatory) turnover rates from the literature (Hunt *et al.* 1987). It must be noted that these values were based on observations in temperate climates,

but a correction of rates for temperature affecting all organisms in the same way would mean a linear transformation of all the rates and would not have any effect on the stability properties of the matrices. (In the case of tardigrades and rotifers, where these non-predatory turnover rates were not provided by Hunt *et al.* (1987), we assumed a value of $2y^{-1}$, comparable to the rates of the other primary consumers and carnivores. These values did not affect the stability results).

The community matrices of the soil food webs were obtained following the same procedure of derivation, using published values of observed biomass, diet specifications and physiological parameters (Hendrix *et al.* 1987; Hunt *et al.* 1987; Andrén *et al.* 1990; de Ruiter *et al.* 1993; de Ruiter *et al.* 1995; Neutel *et al.* 2007), and natural turnover rates from the literature (Hunt *et al.* 1987).

Rationale of assumptions on interaction strengths and stability

Linear functional responses

We used Lotka-Volterra type growth equations assuming linear functional responses of the populations. The same analysis could be performed for interactions that incorporate more complex functional responses (see also Supplementary Material in Neutel *et al.* 2002), but we followed this common practice because of a lack of empirical information on such detail.

Relation between λ_d with zero self-damping and the self-damping needed for stability

We measured λ_d in the absence of self-damping of the populations and found that it approximated the value of this self-damping at the point where the system was just stable. The equivalence of these two properties relates to a corollary of the Gerschgorin circle theorem (Gerschgorin 1931). All the eigenvalues lie in the complex plane bounded by circles whose origins consist of the diagonal elements of the matrix, a_{ii}, which for real matrices consist of points along the real axis. An eigenvalue λ_i can be linearly transformed by the amount that must be subtracted from a_{ii} to allow λ_i to be 0 (by $-|\lambda_i - a_{ii}|$). Therefore, the largest eigenvalue, λ_d, in a zero-diagonal matrix measures the

level of self-damping needed for stability. In our matrices, with one non-zero diagonal element, that of detritus, this match between λ_d and the intra-specific competition is not trivial.

Two necessary conditions for stability

There are two necessary conditions for stability in systems (Levins 1974). The first condition for stability states that in a system of n variables the total feedback $F_k < 0$ for all $k \leq n$, where F_k is the total feedback at the kth level of the system, with level referring to the number of elements in a feedback loop, or a combination of feedback loops. In other words, at each level k, negative feedback must outweigh positive feedback. The second condition states that negative feedback of higher levels must not exceed that of lower levels. This condition refers to oscillatory instability caused by long time lags. Such strong negative feedbacks can cause overcorrection and, in this way, lead to instability. In our analysis we focussed only on the first necessary condition, because the analysis of our observed systems revealed that instability in our systems was caused by positive feedback, not by excessive negative feedback. If self-damping was strong enough to balance out the net positive feedback, the systems were stable. Thus, we found that for our observed systems, the prevalence of negative feedback was enough to guarantee stability.

Derivation of key units of feedback in terms of material flux rates

The units of feedback that were found to play a key role in system vulnerability were the 3-link feedbacks relative to the sum of their corresponding 2-link feedbacks in omnivorous structures: $\sqrt[3]{\frac{|\gamma_{ij}\gamma_{jk}\gamma_{ki}+\gamma_{ik}\gamma_{kj}\gamma_{ji}|}{|\gamma_{ij}\gamma_{ji}+\gamma_{jk}\gamma_{kj}+\gamma_{ki}\gamma_{ik}|}}$, where i is the bottom prey, j is the intermediate predator, and k is the omnivore (Fig. S6). These feedbacks can be expressed in terms of material flow parameters, where all the material flow parameters are defined positive:

$$\sqrt[3]{\frac{|\gamma_{ij}\gamma_{jk}\gamma_{ki}+\gamma_{ik}\gamma_{kj}\gamma_{ji}|}{|\gamma_{ij}\gamma_{ji}+\gamma_{ik}\gamma_{ki}+\gamma_{jk}\gamma_{kj}|}} =$$

$$\sqrt[3]{\frac{Q_{ij}Q_{jk}Q_{ki}e_k(1-e_j)}{Q_{ij}^2 e_j M_k + Q_{ik}^2 e_k M_j + Q_{jk}^2 e_k M_i}} =$$

$$\sqrt[3]{\frac{1-e_j}{\frac{Q_{ij}e_jM_k}{Q_{ik}Q_{jk}e_k}+\frac{Q_{ik}M_j}{Q_{ij}Q_{jk}}+\frac{Q_{jk}M_i}{Q_{ij}Q_{ik}}}}$$

A simpler formalisation of this expression is then given by switching numerator and denominator, to give expression (1) in the main text:

$$\left(\frac{\frac{Q_{ij}}{Q_{ik}Q_{jk}}\frac{e_j}{e_k}M_k+\frac{Q_{ik}}{Q_{ij}Q_{jk}}M_j+\frac{Q_{jk}}{Q_{ij}Q_{ik}}M_i}{1-e_j}\right)^{-\frac{1}{3}} .$$

The $\frac{M}{Q}$ ratios, together with the negative power $-\frac{1}{3}$ show that higher consumption mortality (Q) relative to non-consumption mortality (M), and in particular, the predation pressure of the omnivore on its prey populations ($\frac{Q_{ik}}{M_i}$ and $\frac{Q_{jk}}{M_j}$) increases the magnitude of the expression, and thus increases the strength of destabilising feedback.

Trophic group	Antarctic dry tundra		Antarctic wet tundra	
	Biomass (B_j) ($mgDMm^2$)	Feeding rate (Q_j) ($mgDMm^{-2}y^{-1}$)	Biomass (B_j) ($mgDMm^2$)	Feeding rate (Q_j) ($mgDMm^{-2}y^{-1}$)
1.Predatory mites	5.9 (4.7-7.1)	50.2 (26.1-74.3)		
2.Carnivorous nematodes	0.2 (0-1.7)	0.7 (0-6.1)	3.8 (0-12.1)	16.6 (0-53)
3.Carnivorous tadigrades	9.2 (0-20.8)	27.9 (0-69.1)	77.2 (0.2-152)	155.9 (0-349)
4.Omnivorous nematodes	13.1 (5.4-21.0)	232.2 (88.3-376.1)	41.3 (0-102.5)	677.2 (0-1686)
5.Fungivorous nematodes	2 (0.6-3.5)	186.1 (7.2-365)	1.7 (0-4.8)	201.1 (0-589)
6.Bacterivorous nematodes	15 (3.9-26.3)	775.6 (81-1470)	13.8 (0.9-26.8)	721.7 (0-1486)
7.Omnivorous collembola	224.9 (96.7-353.1)	6622 (5261-8011)	55.5 (0-153.7)	1033.33 (767-1300)
8.Omnivorous rotifers	5.3 (0-11.9)	197 (0-787)	0.3 (0-0.7)	6.5 (0-26)
9.Omnivorous tardigrades	12.6 (0-85.7)	409.8 (0-2813)	28.7 (0-166.7)	829.3 (0-4846)
10.Herbivorous-Microbivorous mites	1.7 (0.7-2.7)	275 (101.7-448)		
11.Herbivorous-Microbivorous mites	1 (0-4.5)	158.3 (0-718.9)		
12.Herbivorous-Microbivorous protozoa	1240 (830-1700)	182222 (556-432778)	1660 (1160-2230)	291666.7 (2778-681111)
13.Detritivorous-Microbivorous protozoa	1.5 (0.8-2.5)	16000 (167-37833)		
14.Herbivorous mites	3.5 (0-8.2)	54.4 (0-127.8)		
15.Herbivorous tardigrades	12.6 (0-85.7)	409.8 (0-2813)	28.7 (0-166.7)	829.26 (0-4846)
16.Detritivorous tardigrades	12.6 (0-128.7)	409.8 (0-2813)	28.7 (0-166.7)	829.26 (0-4846)
17.Detritivorous rotifers	6.9 (0-24.5)	256 (0-1024)	24.5 (0-89.4)	530.8 (0-2297)
18.Detritivorous rotifers	13.1 (0-29.5)	486 (0-1945)	9.1 (0-22.2)	197.15 (0-789)
19.Bacteria	50.8 (12.7-152.3)	537300 (273000-729000)	4.42 (1.08-13.23)	695691.05 (340251-990372)
20.Fungi	5.6 (1.4-16.9)	59700 (30333-81000)	0.49 (0.12-1.47)	38200 (18667-54333)
21.Mosses, Lichens and Liverworts	604000 (321912-1071934)		180000 (156000-204000)	
22.Algae	8400(5400-13200)		1200 (500-2100)	
23.Detritus	33500000 (32600000-34400000)		29600000 (27900000-31300000)	

Table S1. Observed biomass and annual feeding rates in the Antarctic dry and wet tundra.

Mean dry (DM) biomass densities, feeding rates for the trophic groups, and their variablity (in parentheses) (Davis 1981). Representative species or taxa of the trophic groups are (with numbers corresponding to the numbers of the trophic groups above): 1. *Gamasellus racovitzai*, 2. *Coomansus gerlachei*, 3. *Macrobiotus furgicer*, 4. *Eudorylaimus sp.*, 5. *Aphelenchoides haguei*, 6. *Plectus antarcticus*, 7. *Cryptopygus antarcticus*, 8. *Monogononta*, 9. *Echiniscus capillatus*, 10. *Eupodes minutus*, 11. *Ereynetes macquariensis*, 12. *Sarcodina*, 13. *Mastigophora*, 14. *Nanorchestes antarcticus*, 15. *Hypsibius dujardini*, 16. *Hypsibius alpinus*, 17. *Adineta*, 18. other *Bdelloidea*, 21. *Polytrichum alpestre* and *Chorisodontium aciphyllum* (mosses in the dry tundra), *Calliergon sarmentosum*, *Calliergidium austro-stramineum* and *Drepanocladus uncinatus* (mosses in the wet tundra), *Cephaloziella varians* and *Barbilophozoia hatchery* (liverworts).

	1	2	3	4	5	6	7	8	9	10	11	12	13	14	15	16	17	18	19	20
1																				
2																				
3																				
4		0.436(0.727)																		
5		0.0693(0.0299)	0.0297(0.0154)	0.0139(0.0114)																
6		0.495(0.243)	0.218(0.125)	0.102(0.092)																
7	0.94																			
8																				
9			0.188(0.259)																	
10	0.04																			
11	0.02																			
12				0.084(0.111)																
13				0.0001																
14																				
15			0.188(0.259)																	
16			0.188(0.259)																	
17								0.118(0.09)												
18			0.188(0.08)					0.216(0.243)												
19				0.359(0.319)		1		0.3	0.3			0.95	0.95							
20				0.04(0.036)	1		0.465(0.26)	0.033	0.033	0.1	0.05									
21				0.198(0.214)					0.33											
22				0.0026(0.0014)			0.12(0.18)	0.333	0.005(0.0022)	0.9	0.95	0.05		1	1					
23				0.2(0.215)			0.414(0.56)		0.33				0.05			1	1	1	1	1

Table S2. Feeding relations and diet proportions in the Antarctic dry and wet tundra.

The numbers in the margins correspond to the row numbers in Table S1, representing the trophic groups. The elements in each column are the proportions in the diet of the consumer (referred to by the column number). The values in parentheses are the diet proportions in the wet tundra where they differ from the dry tundra. Note that some groups and relations are absent in the Antarctic wet tundra. Details on the proportions of different animal, microbial, or plant prey types in the diets of carnivores and omnivores were not always provided by Davis (1981). In that case specific assumptions were made and tested for their effect on system stability (for details see SI Methods).

System	n	s unscaled	s scaled	λ_d $\mathbf{\Gamma_0^D}$	λ_d $\mathbf{\Gamma_0}$	λ_d symmetric	λ_d asymmetric
Antarctic dry tundra	23	0.03679	0.03679	0.0368	0.03721	0.24617	1.06360
Antarctic wet tundra	18	0.25974	0.25974	0.2599	0.2603	0.21008	1.01726
Inferred-flux Antarctic dry tundra	23	0.08033	0.07825	0.07856	0.08142		
Inferred flux Antarctic wet tundra	18	0.24391	0.23693	0.239	0.2458		
Central Plains Experimental Range	17	0.03548	0.03548	0.03548	0.03553	0.36561	1.62961
Horseshoe Bend-conventional tillage$_\dagger$	14	0.00149	0.00001	5x10^{-18}	1x10^{-16}		
Horseshoe Bend-no tillage$_\dagger$	14	0.00112	0.00001	2x10^{-17}	1x10^{-16}		
Lovinkhoeve-conventional farming	18	0.14607	0.14607	0.146	0.1455	0.26029	1.47651
Lovinkhoeve-integratedfarming	19	0.06116	0.06116	0.06116	0.06106	0.26665	1.44299
Kjettslinge-barley without fertiliser	18	0.00889	0.00889	0.00889	0.00948	0.30084	0.66803
Kjettslinge-barley with fertiliser	18	0.00965	0.00965	0.00965	0.00966	0.31930	0.75805
Schiermonnikoog 1i	8	0.00018	0.00018	0.00017	0.00020	0.08952	0.60712
Schiermonnikoog 1ii	12	0.00598	0.00598	0.00594	0.00874	0.14794	1.00416
Schiermonnikoog 1iii	8	0.00048	0.00048	0.00048	0.00054	0.07957	0.49517
Schiermonnikoog 1iv	8	0.00029	0.00029	0.00029	0.00033	0.07923	0.53868
Schiermonnikoog 2i$_\dagger$	14	0.27668	0.27668	0.2471	0.0133		
Schiermonnikoog 2ii$_\dagger$	13	0.37399	0.37399	0.3154	0.03108		
Schiermonnikoog 2iii$_\dagger$	14	0.25891	0.25891	0.2346	0.01085		
Schiermonnikoog 2iv$_\dagger$	14	0.13398	0.13398	0.1254	0.00838		
Schiermonnikoog 3i	14	0.00921	0.00921	0.00915	0.00912	0.22390	1.23191
Schiermonnikoog 3ii	15	0.01257	0.01257	0.01256	0.01254	0.28043	1.42507
Schiermonnikoog 3iii	14	0.01067	0.01067	0.0106	0.00749	0.24168	1.21259
Schiermonnikoog 3iv	14	0.03531	0.03531	0.03374	0.01856	0.21839	1.22489
Schiermonnikoog 4i	14	0.31664	0.31664	0.3171	0.3191	0.27071	1.28723
Schiermonnikoog 4ii	16	0.13924	0.13924	0.1392	0.1388	0.30606	1.52168
Schiermonnikoog 4iii	17	0.22219	0.22219	0.2226	0.2255	0.32366	1.50409
Schiermonnikoog 4iv	15	0.20432	0.20432	0.2044	0.2053	0.30894	1.45803
Hulshorsterzand 1i$_\dagger$	10	0.00006	0.00004	0.00003	0.04372		
Hulshorsterzand 1ii$_\dagger$	10	0.00009	0.00006	0.00005	0.04642		
Hulshorsterzand 1iii$_\dagger$	10	0.00002	0.00002	0.00001	0.02028		
Hulshorsterzand 1iv$_\dagger$	9	0.00008	0.00004	0.00003	0.1162		
Hulshorsterzand 2i$_\dagger$	13	0.3583	0.3583	0.3051	0.05027		
Hulshorsterzand 2ii$_\dagger$	14	0.25007	0.25007	0.2226	0.0212		
Hulshorsterzand 2iii$_\dagger$	13	0.30856	0.30856	0.2685	0.04997		
Hulshorsterzand 2iv$_\dagger$	14	0.28909	0.28909	0.2557	0.02637		
Hulshorsterzand 3i$_\dagger$	15	0.23471	0.23471	0.2147	0.05939		
Hulshorsterzand 3ii$_\dagger$	14	0.17713	0.17713	0.1668	0.03336		
Hulshorsterzand 3iii$_\dagger$	14	0.12328	0.12328	0.1185	0.01766		
Hulshorsterzand 3iv$_\dagger$	14	0.23965	0.23965	0.2171	0.04198		
Hulshorsterzand 4i	14	0.20374	0.20374	0.2046	0.212	0.29871	1.41952
Hulshorsterzand 4ii	14	0.41934	0.41934	0.4212	0.4291	0.28249	1.41262
Hulshorsterzand 4iii	16	0.45877	0.45877	0.4589	0.4593	0.30675	1.51759
Hulshorsterzand 4iv	15	0.28411	0.28411	0.2863	0.2984	0.30576	1.44353

Table S3. Ecosystem stability measurements of the Antarctic dry and wet tundra and 39 soil food webs.

System size n (number of trophic groups), minimum diagonal strength s before and after scaling, maximum eigenvalue λ_d for the system with detritus $\mathbf{\Gamma_0^D}$, without detritus $\mathbf{\Gamma_0}$, and for the $\mathbf{\Gamma_0}$ matrices where the predator-prey interaction strengths are parametrised from symmetric and asymmetric intervals. Systems in which detritus interactions largely affected λ_d are marked with a † in the table.

The 39 soil food webs are from native and agricultural soils in the USA (Central Plains Experimental Range, Colorado (Hunt *et al.* 1987) and Horseshoe Bend, Georgia (Hendrix *et al.* 1987)), Sweden (Kjettslinge (Andrén *et al.* 1990)) and The Netherlands (Lovinkhoeve farm, Marknesse (de Ruiter *et al.* 1993) and sand-dune successions on the Waddensea island of Schiermonnikoog (Neutel *et al.* 2007) and on the Veluwe, central Netherlands (Hulshorsterzand) (Neutel *et al.* 2007)). The Dutch succession series each represent four stages of early vegetation succession ranging from bare soil to forest.

Stability s is measured as the multiplier of the diagonal elements (only those representing the competition within the populations) of the community matrix for which the dominant eigenvalue lies just below zero (Neutel *et al.* 2002). This was for almost all food webs exactly the same prior to (s unscaled) and after scaling (s scaled) of the original community matrices. The inferred-flux Antarctic dry and wet tundra showed a small difference between scaled and unscaled s, and some of the most stable webs, the Horseshoe Bend systems and Hulshorsterzand 1 systems, showed a relatively big difference, but very small in absolute value. The maximum eigenvalue λ_d closely approximated s scaled.

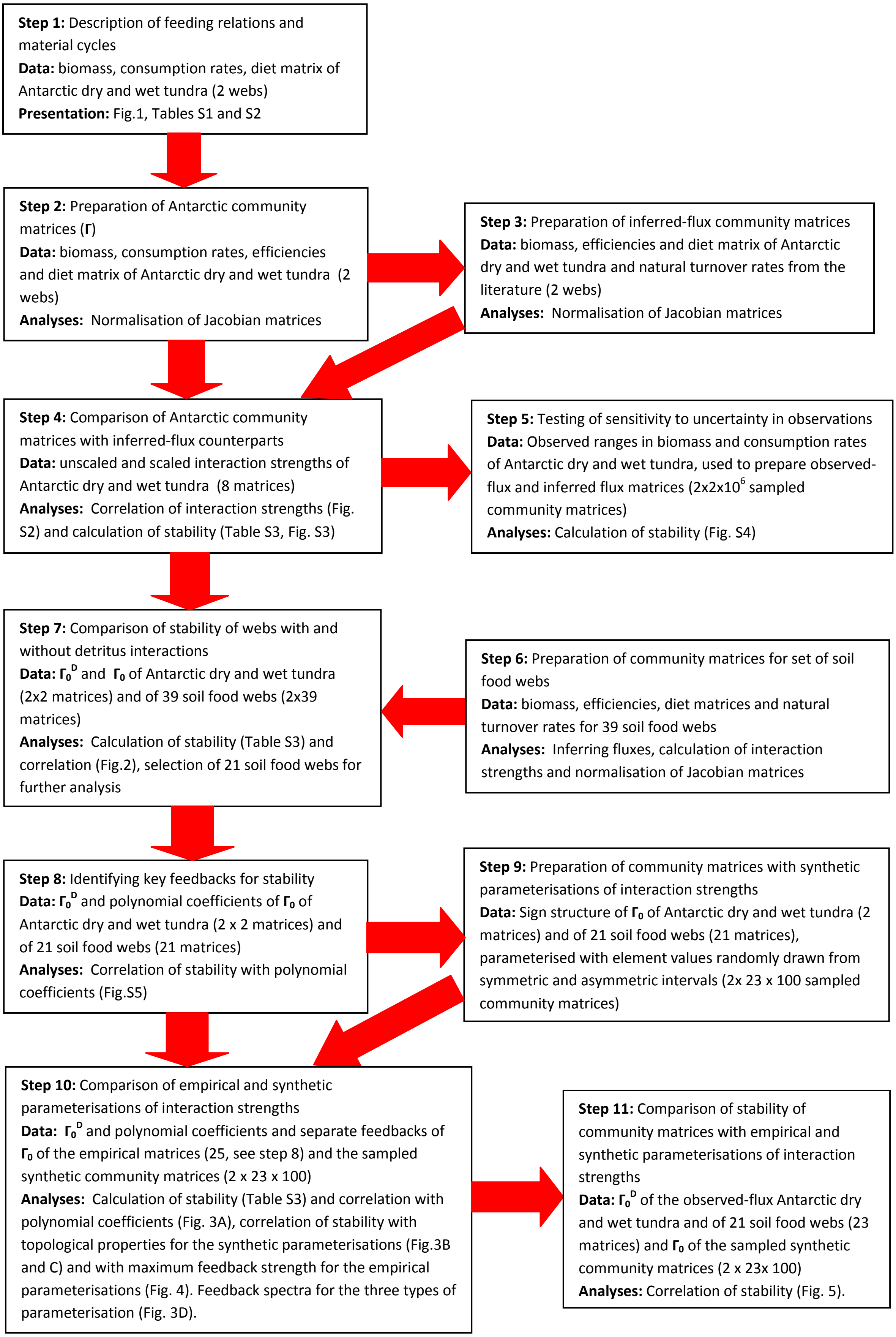

Figure S1. Schematic of the analysis.

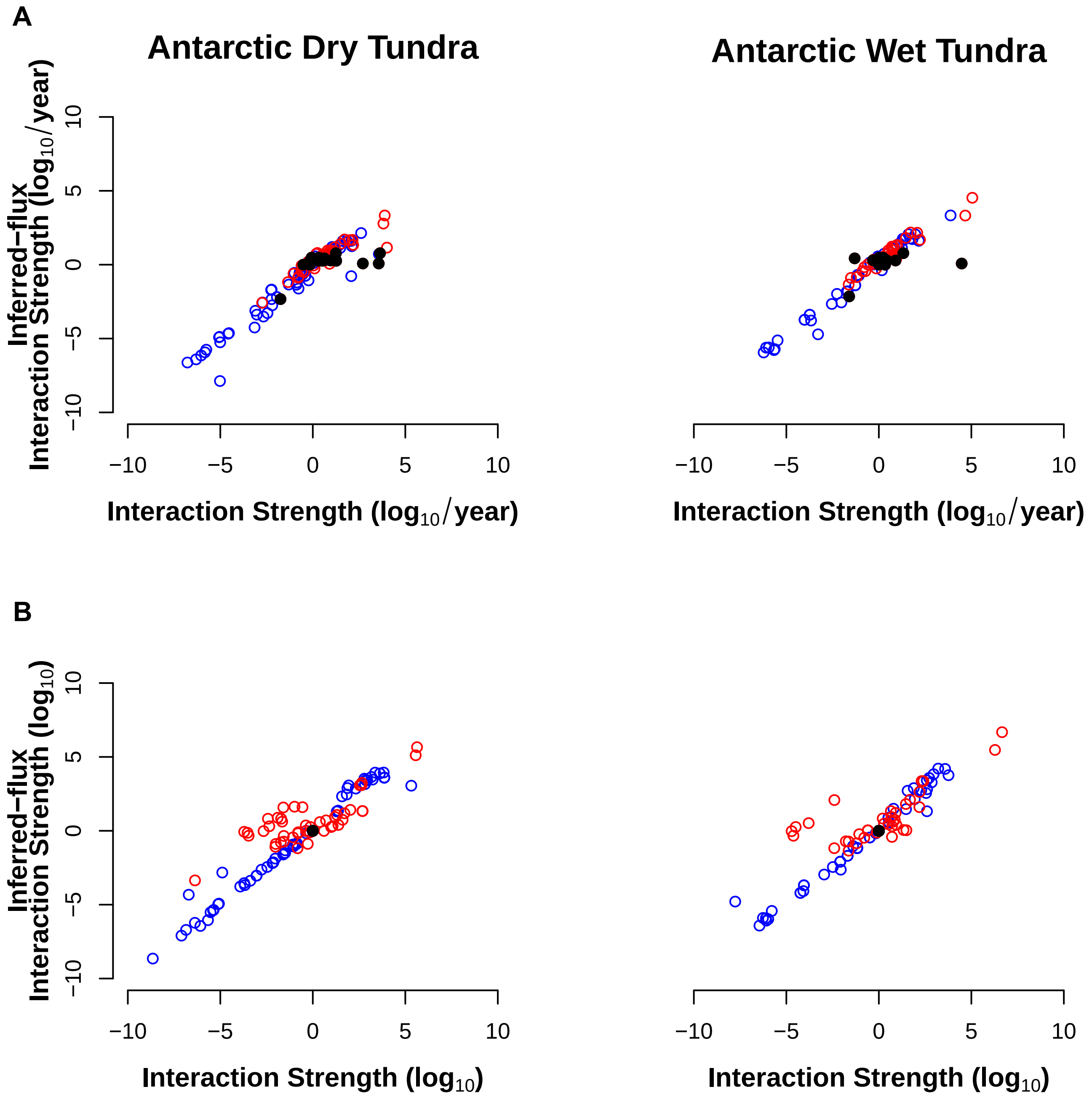

Figure S2. Interaction strengths of the Antarctic dry and wet tundra ecosystems and their inferred-flux counterparts, before and after normalisation.

(A) Comparison of the elements of the Jacobian matrices (absolute values) calculated from the independently measured fluxes with those calculated from inferred-fluxes (dry: N=161, $R^2 = 0.85, P < 10^{-15}$; wet: N=125, $R^2 = 0.87, P < 10^{-15}$) and (B) the same comparison with

elements of the normalised matrices (dry: N=161, $R^2 = 0.87, P < 10^{-15}$; wet: N=125, $R^2 = 0.83, P < 10^{-15}$). Off-diagonal elements are the negative inter-specific interaction strengths (red open circles) and positive inter-specific interaction strengths (blue open circles). Diagonal elements are the negative intra-specific strengths (black solid circles).

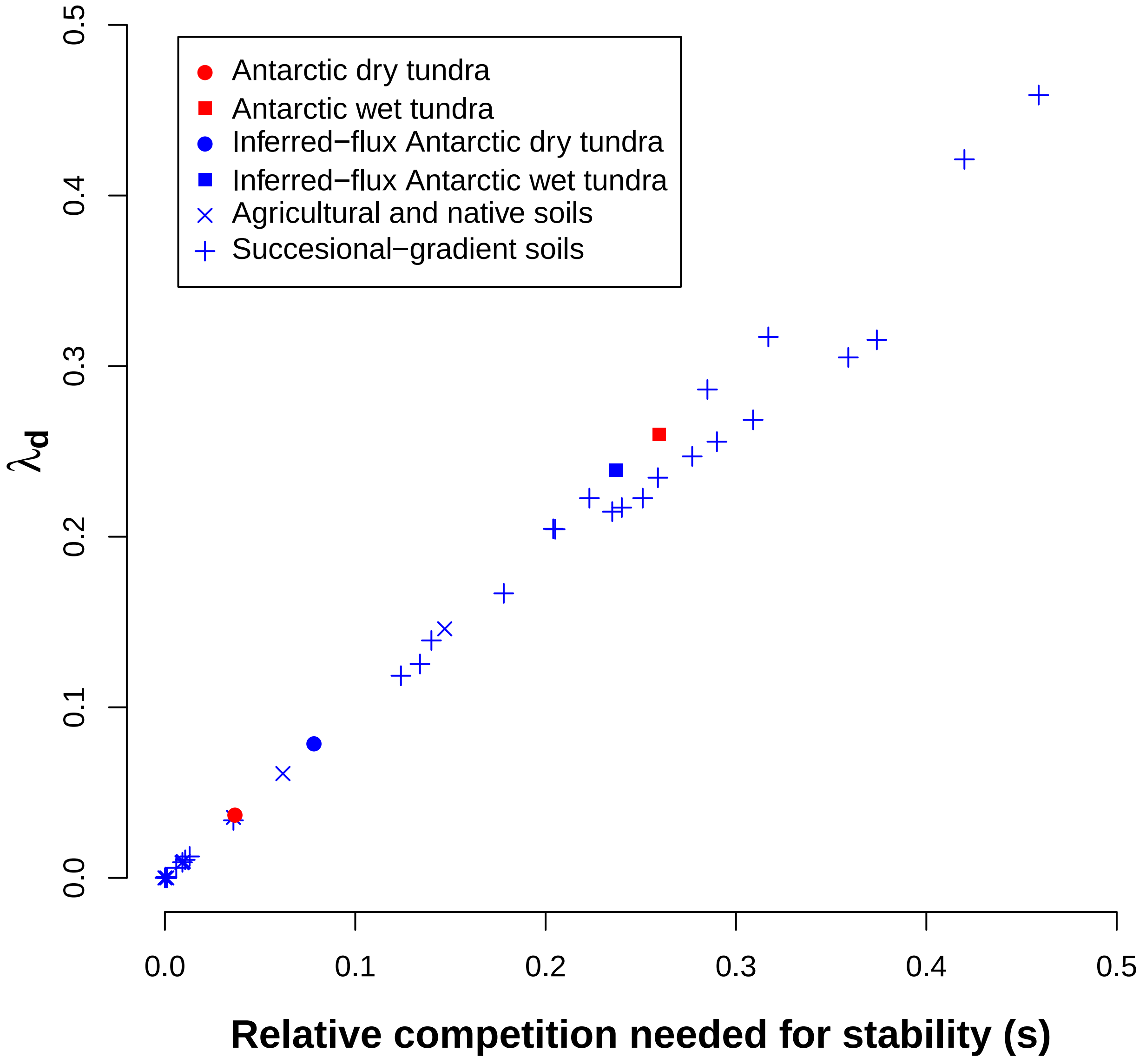

Figure S3. Vulnerability and the critical level of competition.

Relation between the maximum eigenvalue λ_d of the scaled matrices with zero intra-specific interaction of the populations and the relative competition s needed for stability (Neutel *et al.* 2002), for the observed Antarctic dry and wet tundra, their derived counterparts, seven agricultural and native soils (Hendrix *et al.* 1987; Hunt *et al.* 1987; Andrén *et al.* 1990; de Ruiter *et al.* 1993; de Ruiter *et al.* 1995) and 32 soils from two successional gradients (Neutel *et al.* 2007) (see Table S3)

(N=43, $R^2 = 0.99, P < 10^{-15}$).

The figure shows that λ_d of the Antarctic webs, and most of the soil systems, had the same or almost the same value as the critical intraspecific competition level s. Interestingly, in some systems λ_d does not exactly reflect the relative level of competition needed for stability. This showed an impact of the fixed detritus self-damping. Although this deviation was fairly small, and λ_d was still an indicator of the competition needed for stability, it pointed to a more important property. These turned out to be also the systems in which detritus interactions largely affected system vulnerability, as shown by the difference between λ_d of the whole system, with detritus interactions, and that of the same system without detritus interactions (Fig. 2).

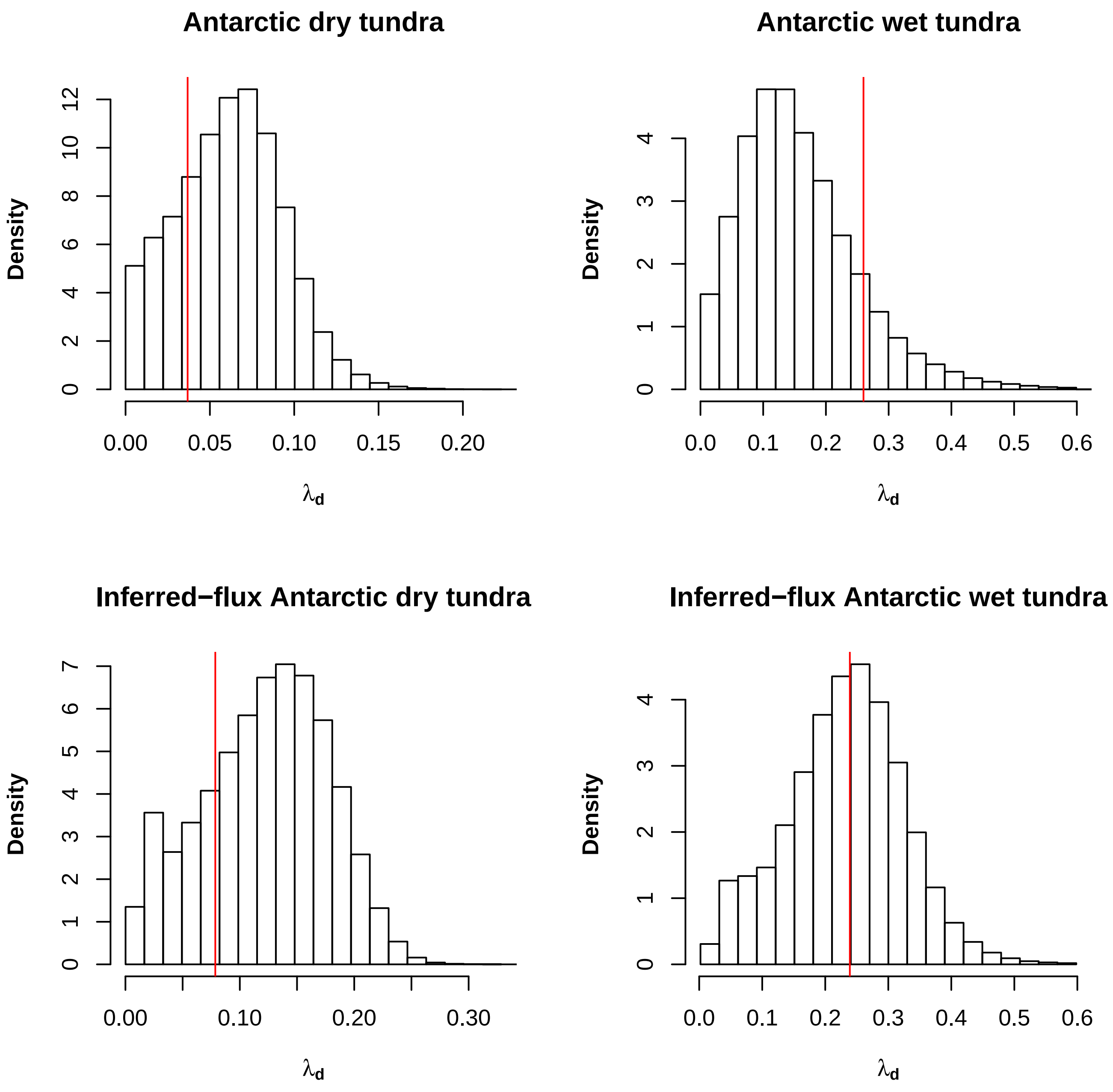

Figure S4. Perturbation of biomass and feeding rates based on the observed variability.

To test the sensitivity of the Antarctic tundra systems to input parameters we sampled biomass and feeding rates randomly from the observed ranges (Davis 1981) (see Table S1), estimated the interaction strengths of these perturbed webs in the same way as for the observed webs, and then calculated their vulnerability (top). We compared these perturbations to those of their inferred-flux counterparts (below).

For each parameter a normal distribution around the mean value (see Table S1) was selected with the given range defining a cut-off. The resulting frequency distributions of λ_d show the sensitivity of the stability results for the mean biomass and feeding rate values (indicated by the red lines) to perturbation, and were based on 10^6 iterations.

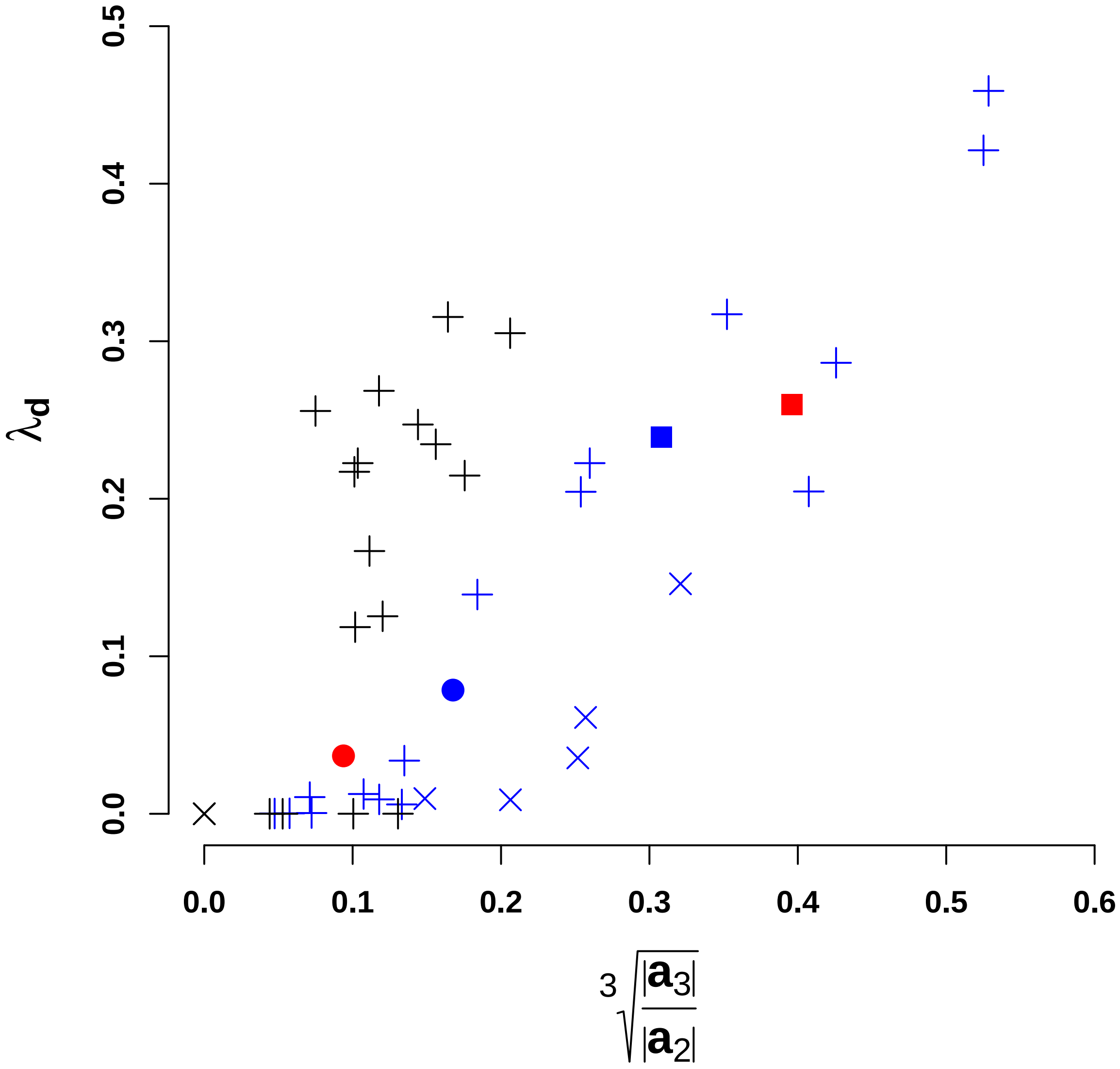

Figure S5. Vulnerability and predator-prey feedbacks.

Relation between system vulnerability λ_d of the $\mathbf{\Gamma_0^D}$ matrices and $\sqrt[3]{\frac{|a_3|}{|a_2|}}$ where a_2 and a_3 are the coefficients of the x^{n-2} and x^{n-3} terms respectively, in the characteristic polynomial of the $\mathbf{\Gamma_0}$ matrices, where n is the size of the system. For the legend see Figure S3. In this figure we have have also added (shown in black) the 18 soil food webs where detritus interactions affected system stability. Regression analysis without the detritus-influenced webs: N=25, R^2=0.84, P< 10^{-10} (and

including the detritus-influenced webs: N=43, R^2=0.47, P$< 10^{-06}$). Note that the systems that were influenced by detritus did not correlate well with $\sqrt[3]{\frac{|a_3|}{|a_2|}}$ only because of the impact of detritus feedbacks on stability. If detritus feedbacks were removed from these systems, the resulting predatory-prey systems would have a λ_d that corresponded to $\sqrt[3]{\frac{|a_3|}{|a_2|}}$.

References

Andrén, Lindberg, T., Bostrm, U., Clarholm, M., Hansson, A.C., Johansson, G. *et al.* (1990) Organic Carbon and Nitrogen Flows. *Ecological Bulletins*, 40, 85-126.

Bokhorst, S.F. (2007) *Functioning of terrestrial ecosystems of the Maritime Antarctic in a warmer climate.* Dissertation. Faculty of Earth and Life Sciences, Free University of Amsterdam, The Netherlands.

Davis, R.C. (1981) Structure and function of two Antarctic terrestrial moss communities. *Ecol. Monogr.*, 5, 125-143.

de Ruiter, P.C., van Veen, J.A., Moore, J.C., Brussaard, L. & Hunt, H.W. (1993) Calculation of nitrogen mineralization in soil food webs. *Plant Soil*, 157, 263-273.

de Ruiter, P.C., Neutel, A.M. & Moore, J.C. (1995) Energetics, patterns of interaction strengths, and stability in real ecosystems. *Science*, 269, 1257-1260.

Gerschgorin, S. (1931) Über die Abgrenzung der Eigenwerte einer Matrix. *Izv. Akad. Nauk. USSR Otd. Fiz.-Mat.*, 6, 749754.

Goddard, D.G. (1977a) *Ecological studies on the terrestrial Acari of Signy Island, South Orkney Islands, in the maritime Antarctic.* Dissertation. University of Leicester, Leicester, England.

Goddard, D.G. (1977b) The Signy Island terrestrial reference sites. VI. Oxygen uptake of *Gamasellus racovitsai* (Trouessart) (Acari: Mesostigmata). *British Antarctic Survey Bulletin*, 45, 1-11.

Goddard, D.G. (1977c) The Signy Island terrestrial reference sites. VIII. Oxygen uptake of some Antarctic prostigmatid mites (Acari: Prostigmata). *British Antarctic Survey Bulletin*, 45, 101-115.

Heal, O.W. & MacLean, S.F. (1975) Comparative productivity in ecosystems - secondary productivity. In *Unifying concepts in Ecology.* eds. Van Dobben, W.H. & Lowe-McConnell, R.H. Dr. W. Junk, The Hague, Netherlands, pp. 89-108.

Hendrix, P.F., Crossley Jr., D.A., Coleman, D.C., Parmelee, R.W. & Beare, M.H. (1987) Carbon dynamics in soil microbes and fauna in conventional and no-tillage agroecosystems. *INTECOL Bulletin*, 15, 59-63.

Hunt, H.W., Coleman, D.C., Ingham, E.R., Ingham, R.E., Elliot, E.T., Moore, J.C. *et al.* (1987) The detrital food web in a shortgrass prairie. *Biol. Fertil. Soils*, 3, 57-68.

Jennings, P.G. (1975) The Signy Island terrestrial reference sites. V. Oxygen uptake of *Macrobiotus furciger* J. Murray (Tardigrada). *British Antarctic Survey Bulletin*, 41 & 42, 161-168.

Jennings, P.G. (1976) *Ecological studies on Antarctic tardigrades and rotifers.* Dissertation. University of Leicester, Leicester, England.

Levins, R. (1974) The qualitative analysis of partially specified systems *Ann. N Y Acad. Sci.*, 231, 123-138.

Maslen, N.R. (1981) The Signy Island terrestrial reference sites. XII. Population ecology of the nematodes with additions to the fauna. *British Antarctic Survey Bulletin*, 53, 57-75.

Neutel, A.M., Heesterbeek, J.A.P. & de Ruiter, P.C. (2002) Stability in real food webs: Weak links in long loops. *Science*, 296, 1120-1123.

Neutel, A.M., Heesterbeek, J.A.P., van de Koppel, J., Hoenderboom, G., Vos, A., Kaldeway, C. *et al.* (2007) Reconciling complexity with stability in naturally assembling food webs. *Nature*, 449, 599-602.

Smith, H.G. (1973a) The Signy Island terrestrial reference sites. II. The Protozoa. *British Antarctic Survey Bulletin*, 33 & 34, 83-87.

Smith, H.G. (1973b) *Studies on the terrestrial Protozoa of the maritime Antarctic.* Dissertation. University of Edinburgh, Edinburgh, Scotland.

Tilbrook, P.J. (1973) The Signy Island terrestrial reference sites. I. An introduction. *British Antarctic Survey Bulletin*, 33 & 34, 65-76.

Tilbrook, P.J. (1977) Energy flow through a population of the collembolan *Cryptopygus antarcticus.* In *Adaptations within Antarctic ecosystems. Proceedings of the Third Scientific Committee for Antarctic Research (SCAR) Symposium on Antarctic Biology* ed. Llano G. Gulf Publishing Company, Houston, Texas, USA. pp. 935-946.

Wynn-Williams, D.D. (1979) Techniques used for studying terrestrial microbial ecology in the maritime Antarctic. In *Cold tolerant microbes in spoilage and the environment.* eds. Russell A.D. and Fuller D. Society for Applied Bacteriology Technical Series 13, Academic Press, London, England. pp. 67-81.
